# A boy with blistering of sun-exposed skin and finger shortening: the first case of Variegate Porphyria with a novel mutation in *protoporphyrinogen oxidase* (*PPOX*) gene in Iran: a case report and literature review

**DOI:** 10.1186/s13052-022-01215-8

**Published:** 2022-02-14

**Authors:** Mohammad Vafaee-Shahi, Saeide Ghasemi, Aina Riahi, Zahra Sadr

**Affiliations:** 1 Pediatric Neurology, Pediatric Growth and Development Research, center, Iran University of Medical Sciences, Tehran, Iran; 2 Pediatrics, Ali Asghar Children’s Hospital, Iran University of Medical Sciences, Tehran, Iran; 3 Pediatrician, Rasool Akram Complex Hospital, Iran University of Medical Sciences, Tehran, Iran; 4 Preventive Medicine and Public Health Research Center, Psychosocial Health Research Institute, Community and Family Medicine Department, School of Medicine, Iran University of Medical Sciences, Tehran, Iran

**Keywords:** Variegate Porphyria, *PPOX* gene, skin and neurological symptoms, Homozygote mutation, Seizure

## Abstract

Variegate Porphyria (VP) is an inherited rare disorder that is caused by mutations in the protoporphyrinogen oxidase (*PPOX*) gene. This deficiency is associated with the accumulation of porphyrins and porphyrin precursors in the body, which, in turn, can potentially result in a variety of skin and neurological symptoms. Here, we reported a 7-year-old boy with homozygous VP and novel mutation on *PPOX* gene. He was admitted with three episodes of generalized tonic-clonic seizure in the last 6 months. He was presented with lesions, hyperpigmentation, fragility, and blistering of sun-exposed skin. The weakness of limbs and brachydactyly were observed. In the follow-up, he had aggressive behavior, learning disability and abdominal pain, particularly around the navel. Eventually, the whole exome sequencing (WES) result reported a novel homozygous pathogenic variant (c.1072G > A p.G358R) in *PPOX* gene which confirmed the VP. He had been advised to be away from the sun and use sunscreen regularly.

## Introduction

The porphyria are a group of genetic rare metabolic disorders which are characterized with a wide range of clinical symptoms based on specific subtype [1]. This disease is associated with overproduction of porphyrins due to genetic mutations of enzymes involved in heme biosynthetic pathway. Variegate porphyria (VP) is one of a group of porphyrias caused by mutations in protoporphyrinogen oxidase (*PPOX*) gene [2]. The typical prevalence of VP is 0.5 per 100,000; however, its prevalence in South Africa is as high as 3 per 1000 [3]. This defect is associated with accumulation of porphyrin or its precursors and results in a variety of symptoms that vary from one person to another [4]. Patients may present with skin and neurological symptoms. Common cutaneous symptoms include fragility and blistering of sun-exposed skin, but vomiting, nausea, constipation, abdominal pain, anxiety, restlessness and seizures, as well as pain and weakness are the most common neurological symptoms [5]. Studies show that various *PPOX* gene mutations are responsible for VP in different families [6]. Although the genetic mutation of *PPOX* is inherited as an autosomal dominant trait, many individuals do not exhibit any symptoms. Although VP incidence is very rare, early diagnosis of different variants and treatment of the disease is important. Here, we report a 7-year-old Iranian boy presented with multiple clinical features and a homozygous pathogenic variant in the *PPOX* gene, c.1072G > A (p.G358R).

## Case presentation

**Fig. 1:**
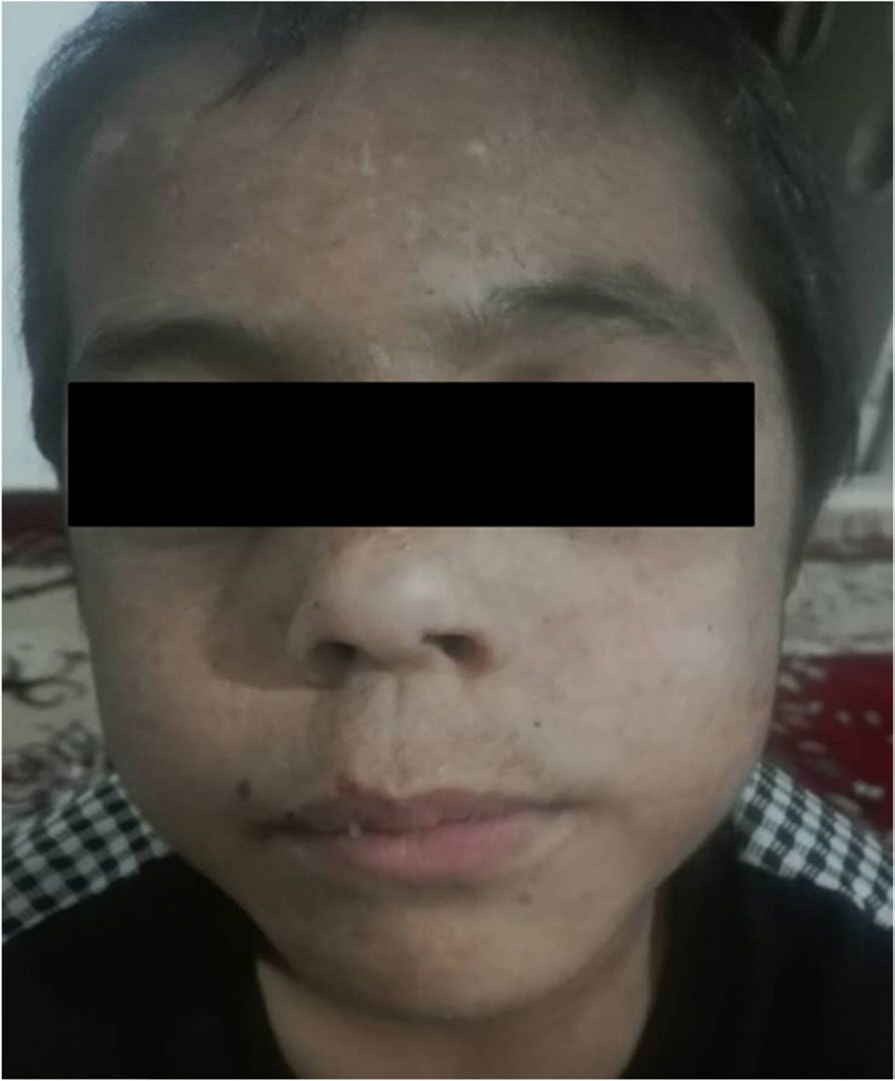
The facial of the patient with coarse and hairy features

**Fig. 2:**
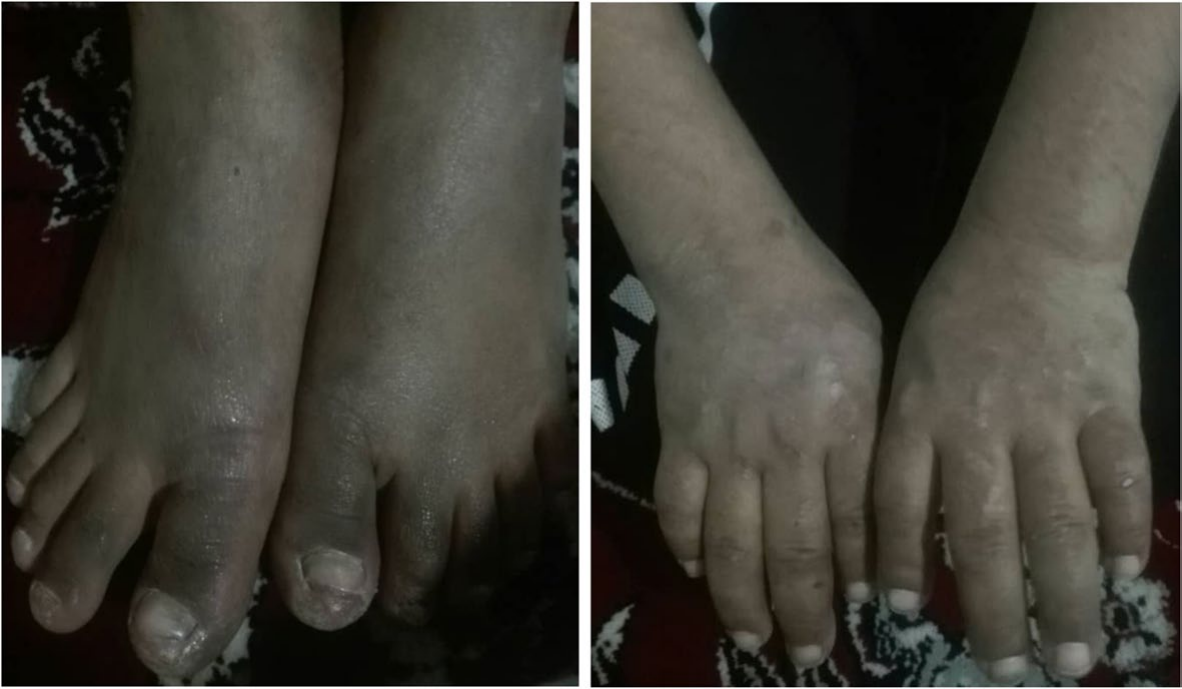
Cutaneous symptoms on hands and feet. Erosive lesions scars, hyperpigmentation, fragility and blistering of sun-exposed skin, and thickened skin were observed

A 7-year-old boy was admitted to our hospital with three episodes of generalized tonic seizure (GTC) in the last 6 months. He didn’t have fever in each episode. His standard laboratory test results were normal without electrolytes imbalance. On physical examination, he was shorter than average. He had coarse and hairy facial features (Fig. 1). Cutaneous symptoms, including erosive lesions scars, hyperpigmentation, fragility and blistering of sun-exposed skin, and thickened skin on hands and feet were observed (Fig. 2). He had presented with weakness of limbs, tremor of the legs while walking and brachydactyly. He was the second child of the family (II/III) from parents with consanguineous marriage. He was born from a normal vaginal delivery method with birth weight of 3 kg. The patient past medical history revealed that he has had developed with skin lesions when he was 2 year old. He was also admitted to a local hospital and received anti-seizure drug due to episodes of generalized tonic-clonic seizure (GTC). She had also a history of delayed developmental milestones and started walking by 3 years old. His first episode of generalized tonic-clonic seizure was occurred when he had 3 years old.

An electroencephalogram (EEG) was performed and revealed abnormal results due to some epileptiform discharges (EDs). The brain magnetic resonance imaging (MRI) reported severe delayed myelination. Differential diagnoses included xeroderma pigmentosum and Cockayane syndrome. The patient had received phenobarbital and seizure was completely controlled. Because of skin lesions, dermatologist recommended a skin biopsy for the possibility of xeroderma pigmentosum. The skin biopsy test result reported the subepidermal bullae compatible with erythropoietic porphyria. Molecular test analysis with whole exome sequencing (WES) was performed to consider any possible mutations. The WES result reported a novel homozygous pathogenic variant (c.1072G > A p.G358R) in *PPOX* gene which indicates the Porphyria Variegate. This pathogenic variant has not been reported previously. In the follow-up, after completing 2 years of treatment, phenobarbital was tapered, but he had developed another episode of status GTC and admitted at hospital. Brian MRI was immediately done and showed normal results. Since it was better not to use phenobarbital for this case, treatment with levetiracetam has been started. To improve skin symptoms, he had been advised to be away from the sun and use sunscreen regularly. He had also aggressive behavior, learning disability and abdominal pain, particularly around the navel.

## Discussion

Variegate porphyria is a very rare and autosomal dominant disorder which is caused by the mutations in protoporphyrinogen oxidase. PPOX is one of the fundamental enzymes responsible for the synthesis of heme, as a main part of hemoglobin and other hemoproteins. Porphyrins are essential for hemoglobin function. When PPOX is deficient, porphyrins are oxidized to protoporphyrin and coproporphyrin, transported in the blood plasma and cause higher sensitivity of the skin to sunlight. VP symptoms usually start in adulthood and vary from person to person (Table 1) [17]. It may be associated with skin symptoms, neurological symptoms or both. Sun sensitivity, blisters, sores and discoloration after sun exposure are the most common type of skin symptoms in patients with VP. Acute attacks are neurological symptoms that may occur as a consequence of exposure to hormonal changes, certain medications and dieting. Abdominal pain, nausea, vomiting, diarrhea and constipation are the other symptoms that a person with VP my experience. Weakness of muscles, seizures, hypertension and increased heart rate may also occur (Table 1). Anxiety and hallucinations have been reported as mental changes [18].

**Table 1:** The most common clinical symptoms of VP reported by previous studies

Cases	Skin symptoms	Other clinical findings	Refs
A 30-year-old woman	Repeated vesicles and brownish pigmentation; brownish pigmentations and crusts in hands and forearms	Weakness and dysesthesia in all limbs; hallucination; urinary disturbance; dark-red urine	[7]
A 9-month girl	Blistering of the face and hands; erosions; crusting; pigmentation and milia; scarring of sun-exposed areas; thickened and infiltrated skin	Elevated erythrocyte protoporphyrin; marked foreshortened; stubby fingers and toes; brachydactyly; delayed motor development;	[8]
A male infant	Blistering and fragility of the skin of hands, face and ears; hypertrichosis; hyperpigmentation; scarring and milia;	Epilepsy; developmental delay; nystagmus and clinodactyly	[8]
A 14-year-old girl	Photosensitivity	Mental retardation; clinodactyly; enhanced erythrocyte protoporphyrin concentration	[9]
A 7-year-old boy	Severe photosensitivity; skin fragility, blistering, erosions and pronounced scarring in sun-exposed body areas	Brachydactyly; raised proto-porphyrin concentrations;	[10]
A 73 years old woman	Cutaneous eruptions to the sun-exposed areas; increased facial pigmentation	Acute abdominal pains and bloating	[11]
A 25-year-old female	Rash; bullous lesions in the light exposed area	Dark coloured urine; nausea and vomiting; afebrile; attack of abdominal pain; constipation	[12]
An Italian case	Photosensitivity; photomutilations;	mental retardation; aphasia and aggressivity	[13]
A 35 years old female	Blister formation after sun exposure;	Reddish urine; abdominal pain, nausea, vomiting and seizures; muscular weakness; Amyloid A amyloidosis	[14]
A 12-year-old female	Blisters	Epilepsy; mental retardation; premature adrenarche; death	[15]
A 23-year-old female	Itchy blisters in face and upper limbs; scarring; erythematous maculopapular lesions; cutaneous porphyria, cutaneous lupus, drug-induced photosensitivity, epidermolysis bullosa acquisita and actinic prurigo	Excess protoporphyrin and coproporphyrin in stool	
A 36-year-old woman	cutaneous manifestations; photosensitivity	Elevated erythrocyte protoporphyrin	[16]
A 24-year-old female	Blisters; superficial erosions; haemorrhagic crusts, milia and patchy hyper pigmentation of the extensor sites of fingers and hands; skin fragility	No neurological symptoms; enhanced urinary coproporphyrin; faecal protoporphyrin excretion; decrease of porphobilinogen deaminase activity; dark urine; coproporphyrin and faecal protoporphyrin excretion	

To the best of our knowledge, around 15 cases with homozygous VP have been reported worldwide. Here, we reported the first case of homozygous VP with novel mutation on *PPOX* gene in Iran. Our case had a history of GTC episodes with the history of developmental delay. Similarly, Hif et al., [8] reported a VP case who developed epilepsy at 5 months and developmental delay. Our case had a coarse and hairy facial feature. The most common skin symptoms in our patient were erosive lesions scars, hyperpigmentation, fragility and blistering of sun-exposed skin, as well as thickened skin on hands and feet. Weakness of limbs, tremor of the legs while walking and brachydactyly were the other findings in this case. Hif et al., [8] reported blistering and fragility of the face and hands, hyperpigmentation, hypertrichosis, erosions, crusting, pigmentation and milia, scarring of sun-exposed areas, as well as foreshortened, stubby fingers and toes, brachydactyly, delayed motor development as the most common skin and neurological symptoms in two cases with VP. In the follow-up, our case showed aggressivity learning disability. A previous study reported mental retardation in a 14-year-old girl [9]. A more recent study has reported mental retardation and aggressive behavior in an Italian case during a long-term follow up [13]. Shimizu et al., [7] reported a 30-year-old woman with VP who had multiple skin and neurological symptoms. Repeated vesicles and brownish pigmentation on upper extremities were the most common skin symptoms in this case. Moderate weakness, dysesthesia and hyperreflexia in all limbs were the most common neurological findings in this patient. Some studies reported reddish and dark colored urine in patients with VP [12, 14]. Tsuchiya et al., [14] found the existence of amyloid and amyloidosis in a 35-year-old woman with VP which was complicate with end-stage renal failure and gastrointestinal symptoms. A previous study reported a young VP case with epilepsy, mental retardation and premature adrenarche symptoms, but death occurred later [15]. Hepatocellular carcinoma in VP has been described in at least eight cases with VP, indicating the risk of cancer in these patients [19]. All of these data emphasize that there are various skin and neurological symptoms in these patients. There are patients who may have minor symptoms without remarkable clinical manifestations. The severity of VP symptoms feasibly depends on the type of *PPOX* mutations. Therefore, early diagnosis and treatment the clinical features of the disease are important to improve the quality of life among these cases. Some studies reported different missense mutations in the *PPOX* gene which were correlated to VP occurrence. A previous study identified two underlying missense mutations in the *PPOX* gene, consisting of a G-to-A transition in exon 6 (G169E), and a G-to-A transition in exon 10 (G358R), which were correlated to homozygous VP [17]. In another study, Roberts et al., [20] reported two missense mutations (D349A and A433P) in *PPOX* gene which may be correlated to VP without clinical penetrance in heterozygotes. Poblete-Gutierrez et al., [10] found two different mutations in exon 7 and exon 13 of the *PPOX* gene in a 7-year-old boy. More recently, Bonuglia et al., [13] reported a novel mutation in *PPOX* gene (1061C > T/397G > A) in an Italian case. Another study showed two novel mutations in *PPOX* gene (c. 657–658 insertion and IVS 11–1 G → A transition) in a 36-year-old woman with VP [16]. Therefore, these findings emphasize the importance of molecular genetic testing to find any mutations in *PPOX* gene and their correlation to VP occurrence and disease severity.

More importantly, the genetic basis of the disease should be considered. Although no evidence of similar conditions was reported in our patient’s family, he was born from parents with consanguineous marriage. Therefore, elucidation of the genetic basis in this family is important for genetic counselling. Moreover, each child of an individual with VP has a 50% chance of inheriting the mutation [11]. Offspring who inherit the *PPOX* mutation may be more or less severely affected than their parent. Therefore, we believe that the case presented herein under-lines the overall importance of genetic studies in the porphyrias.

## Conclusion

Here, we reported the first Iranian case of homozygous VP with a novel mutation on *PPOX* gene. Various skin and neurological symptoms may be found in these patients, which are probably due to different mutations in *PPOX* genes. Therefore, further studies are necessary to evaluate the relationship between genotype and phenotype of the disease. The VP diagnosis is firstly depends on clinical findings, especially skin and neurological symptoms. Once a case is suspected of having VP, extra laboratory tests on urine, blood and stool can be performed. As most studies reported elevated protoporphyrin levels in cases of homozygous VP, measurement of protoporphyrin or coproporphyrin in the blood, urine and stool is helpful. However, the most sensitive screening test is the assessment of plasma porphyrin. Genetic testing of *PPOX* gene is valuable to confirm the disease diagnosis. Interestingly, our patient was born from parents with consanguineous marriage; however, no evidence of similar conditions was reported in his family. Since consanguineous marriage is common in Iran, there may be a relationship between the incidence of VP and consanguineous marriage. Therefore, further considerations are required to evaluate the correlation. Therefore, premarital genetic counseling and education may be helpful. Avoiding excess sun exposure can reduce the blisters and skin lesions in these cases.

## Data Availability

The datasets used during the current case study are in the manuscript.
